# Noninvasive sublingual microvascular imaging reveals sex‐specific reduction in glycocalyx barrier properties in patients with coronary artery disease

**DOI:** 10.14814/phy2.14351

**Published:** 2020-01-20

**Authors:** Judith Brands, Carl A. Hubel, Andrew Althouse, Steven E. Reis, John J. Pacella

**Affiliations:** ^1^ Magee‐Womens Research Institute Pittsburgh PA USA; ^2^ Department of Obstetrics, Gynecology and Reproductive Sciences University of Pittsburgh Pittsburgh PA USA; ^3^ Division of General Internal Medicine University of Pittsburgh Pittsburgh PA USA; ^4^ Division of Cardiology University of Pittsburgh Pittsburgh PA USA

**Keywords:** coronary artery disease, endothelial glycocalyx, imaging, microvascular dysfunction, sex differences

## Abstract

**Background:**

Risk factors for coronary artery disease (CAD) have been associated with endothelial dysfunction and degradation of the endothelial glycocalyx. This study was designed to compare sublingual microvascular perfusion and glycocalyx barrier properties in CAD patients and controls using noninvasive side stream darkfield imaging.

**Methods:**

Imaging of the sublingual microvasculature was performed in 52 case subjects (CAD confirmed by left heart catheterization) and 63 controls (low Framingham risk score). Red blood cell (RBC) filling percentage and functional microvascular density, measures of microvascular perfusion, and perfused boundary region (PBR), an index of glycocalyx barrier function, were measured in microvessels with a diameter ranging from 5–25 µm.

**Results:**

RBC filling percentage was lower in patients with CAD compared to controls (*p* < .001). Functional microvascular density did not differ between groups. The overall PBR was marginally greater in the CAD group compared to the control group (*p* = .08). PBR did not differ between male CAD cases and controls (*p* = .17). However, PBR was greater in females with CAD compared with female controls (*p* = .04), indicating reduced glycocalyx barrier function. This difference became more pronounced after adjusting for potential confounders.

**Conclusions:**

Our data suggest that patients with CAD are characterized by a reduction in percentage of time microvessels are occupied by RBCs. In addition, CAD is significantly associated with impaired sublingual microvascular glycocalyx barrier function in women but not men. More research is needed to determine the significance of peripheral microvascular dysfunction in the pathophysiology of CAD, and how this may differ by sex.

## Graphical abstract

We found that both males and females with coronary artery disease (CAD) have significantly impaired sublingual microvascular perfusion reflecting a reduced microvascular tube hematocrit. Furthermore, females with CAD exhibited a significant reduction in glycocalyx barrier properties compared to female controls, whereas males with CAD showed no difference in glycocalyx barrier function, suggesting a sex‐specific reduction in glycocalyx barrier properties.

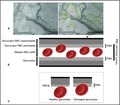

## 1 INTRODUCTION

Coronary artery disease (CAD) in humans develops over decades and can begin early in life. CAD is clinically diagnosed by an electrocardiogram, echocardiogram, stress testing with myocardial perfusion imaging, or cardiac catheterization, all of which detect CAD only when advanced enough to cause ischemia or angiographic luminal narrowing. Early identification of CAD, and treatment of the disease in patients to prevent progression and clinical events, improves prognoses. Coronary or peripheral vascular endothelial dysfunction is one of the first recognizable signs of CAD development and has been shown to be an independent predictor of cardiovascular events among patients with established CAD (Gori, 2018; Matsuzawa & Lerman, 2014). Noninvasive assessments have potential utility in further elucidating the nature of endothelial dysfunction among patients with CAD, and in guiding treatment and improving outcomes.

The endothelial glycocalyx lines the luminal side of the vascular endothelium, in direct contact with flowing blood. It is structurally composed of proteoglycans, glycosaminoglycans, glycoproteins, and glycolipids. It regulates vascular permeability and blood cell–vessel wall interactions, mediates shear stress sensing, contributes to homeostatic signaling, and therefore fulfills a vasculoprotective role (van Berg, Vink, & Spaan, 2003; Florian et al., 2003; Henry & Duling, 1999; Huxley & Williams, 2000; Mochizuki et al., 2003; Pahakis, Kosky, Dull, & Tarbell, 2007). Several risk factors for CAD (e.g., hyperglycemia, diabetes, and pro‐inflammatory cytokines) have been associated with a degraded endothelial glycocalyx, as demonstrated in both animal and human studies (van Berg, Spaan, Rolf, & Vink, 2006; Constantinescu, Vink, & Spaan, 2003; Henry & Duling, 2000; Nieuwdorp, Haeften, et al., 2006; Nieuwdorp, Mooij, et al., 2006). However, the glycocalyx is difficult to study due to its high vulnerability to damage ex vivo. Recently, a noninvasive method using side stream darkfield (SDF) imaging became available for accessing the glycocalyx. SDF video image analysis with automated data capture is one of the principal methods to assess microvascular health noninvasively in vivo. This instrument measures the degree to which red blood cells (RBC) access (radially penetrate) the sublingual microvascular glycocalyx, and is based on the concept that RBCs can penetrate deeper toward the endothelium when the glycocalyx is damaged or unstable (functionally thinner) (Dane et al., 2015; Ikonomidis et al., 2017; Jaarsma et al., 2017; Lee et al., 2014; Mulders, Nieuwdorp, Stroes, Vink, & Pinto‐Sietsma, 2013; Xue, Jiang, Chen, & Chen, 2018). This RBC accessible portion of the glycocalyx is commonly termed the perfused boundary region (PBR). A variety of cardiovascular diseases or risk factors, including end‐stage renal disease (Vlahu et al., 2012), lacunar stroke (Martens, Vink, Oostenbrugge, & Staals, 2013), ischemic heart disease (Gorshkov, Klimushina, Boytsov, Kots, & Gumanova, 2018), and systemic sclerosis (Machin, Gates, Vink, Frech, & Donato, 2017), have been associated with an increase of the sublingual microvascular PBR.

Jaarsma and coworkers used SDF to show a significant increase in sublingual microvascular PBR in patients with microvascular angina (angina pectoris, ST depression on treadmill testing, normal coronary angiogram) compared with controls (without history of chest pain, documented coronary artery disease, or myocardial infarction), suggesting deeper erythrocyte penetration into the glycocalyx. However, a considerably wider variation in PBR's was observed in patients with obstructive CAD such that they did not differ from either controls or patients with angina. The authors suggested that this may reflect heterogeneity of microvascular dysfunction, or even the absence of microvascular dysfunction in a subset of patients with obstructive CAD (Jaarsma et al., 2017). In contrast, Mulders and coworkers found an increased PBR in patients with premature CAD and a family history of premature cardiovascular disease, and their first degree families members with elevated coronary artery calcification (Mulders et al., 2013). However, neither of these studies stratified their data by sex, a variable that could contribute to the variation in PBR observed in patients with obstructive CAD.

We tested the hypothesis that patients with CAD have reductions of endothelial glycocalyx barrier function in the sublingual microvasculature when compared to age‐matched controls with low cardiovascular risk scores, and in consideration of potential heterogeneity, tested for difference by sex among CAD patients and controls (Miranda, Carvalho, Schmidt, Marin‐Neto, & Pazin‐Filho, 2016). SDF imaging also enabled us to likewise compare functional (RBC‐perfused) microvascular density and the degree to which sublingual microvascular segments are occupied with RBCs (RBC filling percentage) as indices of perfusion (Lee et al., 2014; Machin et al., 2017).

## 2 MATERIALS AND METHODS

### 2.1 Study population

The University of Pittsburgh Institutional Review Board approved the study, and all study participants provided written informed consent. Patients suspected to have cardiovascular disease and evaluated by left heart catheterization at the UPMC Presbyterian University Hospital (Pittsburgh, PA) were prospectively enrolled. Patients with a previous heart transplant or those undergoing chemotherapy or anti‐inflammatory therapy (such as for cancer or for a rheumatologic disorder) were excluded. We enrolled 56 CAD case study subjects at the catheterization laboratory holding area. An additional seven subjects were enrolled after hospital admission. Subjects within this group without evidence of any angiographic abnormalities or with incomplete recordings were excluded for further analysis (*n* = 11). The 52 patients in whom CAD was diagnosed (Table 1), had angiographic abnormalities ranging from luminal irregularities to multivessel severe obstructive CAD. We interrogated the sublingual microvasculature noninvasively at the time of study participant informed consent and enrollment. Baseline characteristics were obtained from physician‐provided medical records. Diagnoses of hyperlipidemia or hypertension were based on physician notes in patient history. Controls (*n* = 63) were selected from the Heart Strategies Concentrating on Risk Evaluation (Heart SCORE) study, a community‐based, prospective cohort study in southwestern Pennsylvania (Aiyer et al., 2007; Bambs et al., 2011; Mulukutla et al., 2010; Olafiranye et al., 2015). Exclusion criteria included history of myocardial infarction, known CAD, uncontrolled hypertension, peripheral vascular disease, ejection fraction <50%, valvular heart disease, significant endocrine, hepatic, renal or inflammatory disease, and surgery or major trauma in the previous month. Control group eligibility criteria included classification as low cardiovascular risk using the Framingham Risk Score criteria (below 10% risk of a coronary heart disease event in the next 10 years) (Hermansson & Kahan, 2018; Wolfson et al., 2017). Potential participants were approached at the time of their Heart SCORE study visit. For all study participants, sublingual measurements were performed at the University of Pittsburgh Medical Center Heart and Vascular Institute after consent and enrollment.

**Table 1:** Descriptive characteristics of study patients

	Control	CAD	*p*‐Value
All patients			
# Patients	63	52	
Age	66.4 ± 6.9	69.2 ± 10.7	.09
Females, *n* (%)	41 (65%)	13 (25%)	<.01
Race			<.01
White	39 (62%)	46 (89%)	
Black	23 (37%)	5 (10%)	
BMI	29.7 ± 6.5	29.8 ± 6.1	.92
Hyperlipidemia	24 (38%)	36 (69%)	<.01
Hypertension	21 (33%)	41 (79%)	<.01
Blood pressure			
Systolic	126 ± 12.9	142 ± 27.5	<.01
Diastolic	73.7 ± 8.7	69.6 ± 13.0	.04
Mean	91.0 ± 9.3	93.6 ± 15.5	.28
Males only			
# Patients	22	39	
Age	66.0 ± 7.1	70.2 ± 10.4	.09
Race			.04
White	16 (73%)	35 (90%)	
Black	6 (27%)	3 (8%)	
BMI	29.6 ± 4.6	30.3 ± 6.1	.65
Hyperlipidemia	8 (36%)	27 (69%)	.02
Hypertension	10 (46%)	31 (80%)	.01
Blood pressure			
Systolic	127 ± 15.2	138 ± 22.7	.04
Diastolic	73.7 ± 9.2	69.2 ± 13.1	.15
Mean	91.4 ± 10.6	92.2 ± 13.9	.82
Females only			
# Patients	41	13	
Age	66.6 ± 6.9	66.4 ± 11.5	.92
Race			.08
White	23 (56%)	11 (85%)	
Black	17 (42%)	2 (15%)	
BMI	29.8 ± 7.4	28.4 ± 6.1	.56
Hyperlipidemia	16 (39%)	9 (69%)	.11
Hypertension	11 (27%)	10 (77%)	<.01
Blood pressure			
Systolic	125 ± 11.7	151 ± 37.8	<.01
Diastolic	73.7 ± 8.5	70.8 ± 13.1	.35
Mean	90.8 ± 8.6	97.7 ± 19.5	.08

Note

Continuous variables were compared using two‐sample *t* tests. Categorical variables were compared using Fisher's exact test.

### 2.2 Sublingual microvascular imaging

**Figure 1:**
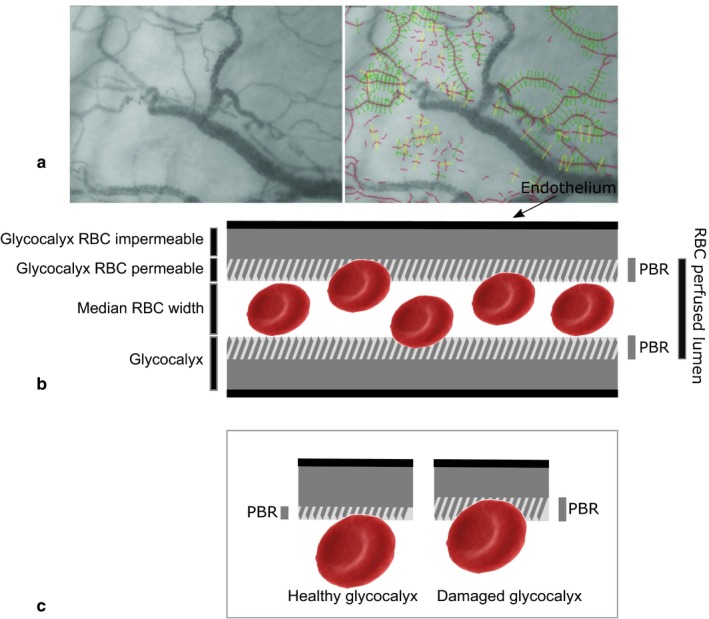
(a). The sublingual microvasculature was imaged using a handheld video microscope to detect the hemoglobin of passing red blood cells (RBCs). The resulting black contrast reveals the RBC perfused lumen of the vessels (left panel). In each recording, the software detects vascular segments (indicated by red lines along the longitudinal axis) every 10 µm along the length of microvessels. After quality control, all valid segments (vascular segment marked with a green line) were used for further analysis, while all other segments (marked yellow) were discarded (right panel). (b) Schematic of a cross section of a microvessel. The endothelial glycocalyx on the luminal side of the endothelium consists of a cell impermeable portion (solid gray) that cannot be accessed and a more cell permeable part (striped) with less limited accessibility for RBCs. The perfused boundary region (PBR) is defined as the difference between the median RBC column width and the maximal RBC column width (width of the RBC perfused lumen), divided by 2. (c) The left panel represents a healthy glycocalyx with greater ability to limit the access of certain molecules, including RBCs, to the endothelial cell membrane. Damage to the glycocalyx (right panel) has been associated with a reduction in the RBC‐impermeable part of the glycocalyx. As a result, RBCs penetrate deeper into the glycocalyx, shifting the outer (solid gray) edge of the RBC perfused lumen toward the endothelium, increasing the PBR

All 115 study subjects underwent imaging of the sublingual microvasculature using a handheld video microscope (MicroVision Medical). Microvascular properties were determined using GlycoCheck Measurement Software (GlycoCheck) as described previously (Dane et al., 2014; Lee et al., 2014). Briefly, a series of 40 frames of digital images were recorded in which approximately 3,000 microvascular segments of RBC column width between 5 and 25 µm were identified. Each segment is 10 µm in length and divided into 21 line markers (0.5 µm apart), resulting in 840 measurement sites (21 line markers in 40 frames) per segment. Segments with an RBC present on at least 60% of the line markers in the first frame were used for further analysis (see Figure 1a). Based on the distribution of RBCs within the vascular segment the maximum and median RBC column width was calculated. The PBR is used as an index of glycocalyx barrier function, wherein a larger PBR indicates a reduction of ability of the glycocalyx to exclude RBCs and, hence, reduced barrier function. The PBR was calculated by taking the difference between portions of the RBC‐perfused lumen, namely the maximal RBC column width minus the median RBC column width, divided by 2 $\left[\text{PBR}=\frac{\text{Maximal RBC column width}-\text{Median RBC column width}}{2}\right]$ (see Figure 1b). The median PBR was calculated for each 10 µm interval within the range of 5–25 µm median RBC column width (“diameter”); these data were then averaged to provide a single overall PBR value describing the 5–25 µm diameter range for each participant. PBR is likewise determined for 5–9 μm, 10–19 μm, and 20–25 μm diameter subclasses. The RBC filling percentage, representing an estimate of longitudinal tube hematocrit of perfused microvascular segments, was calculated as the percentage of time in which a particular vascular segment was occupied by RBCs. RBC filling percentage was reported as the average across microvascular segments of 5–25 µm diameter. Functional microvascular density was determined by the length of vascular segments perfused with RBCs per area of tissue visualized, expressed as micrometer of microvessel length per mm^2^ of area of tissue (µm/mm^2^). This measure of vascular density does not include plasma‐only perfused microvessels.

### 2.3 Statistical analysis

We summarized continuous variables as mean ± *SD* and categorical variables as frequency (percentage). We compared characteristics of participants with and without CAD using two‐sample *t* tests for continuous variables and Fisher's Exact test for categorical variables. We performed a multivariable regression analysis to test the differences between CAD and control participants while adjusting for potential confounders (age, body mass index (BMI), race, hypertension, and hyperlipidemia). All statistical analyses were performed using SAS version 9.4 and JMP Pro version 13.1.1 (SAS Institute).

## 3 RESULTS

### 3.1 Study participant characteristics

Characteristics of study subjects are presented in Table 1. The CAD and control groups did not differ significantly by age or BMI. There were significantly fewer participants of black race in the CAD group (*p* < .01). Fifteen percent of females with CAD were of black race compared to 42% of female controls (*p* = .08). For males, 8% of the CAD and 27% of the controls were of black race (*p* = .04). The CAD group overall had a higher prevalence of hyperlipidemia (*p* < .01) and hypertension (*p* < .001) with higher mean systolic (*p* < .01) and diastolic (*p* = .04) blood pressures. Sixty‐nine percent of males with CAD had hyperlipidemia compared to 36% of male controls (*p* = .02), similar to the prevalence of hyperlipidemia in case and control women (69% vs. 39%, *p* = .11). Hypertension was more common both among men with CAD compared to their controls (80% vs. 46%, *p* = .01) and women with CAD compared to their controls (77% vs. 27%, *p* < .01).

### 3.2 Sublingual microvasculature imaging

**Figure 2:**
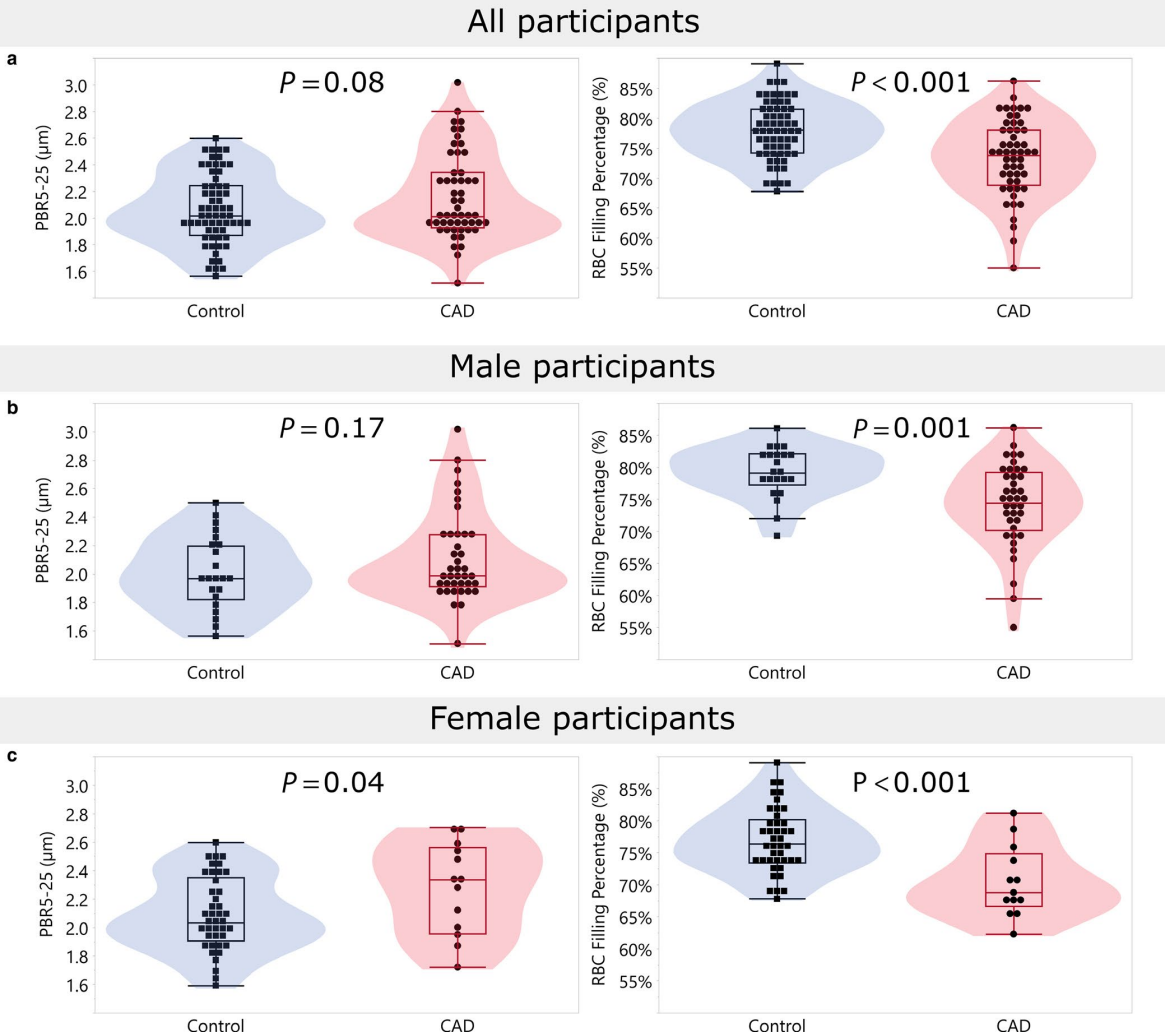
(a) Perfused boundary region (PBR) of the sublingual microvasculature, a measurement of the red blood cell (RBC) permeable aspect of the glycocalyx, measured in controls from the Heart SCORE study (Controls) and subjects with coronary artery disease (CAD). The greater median PBR in subjects with CAD compared to controls was of borderline significance (*p* = .08). (b) RBC filling percentage, a measure of microvascular perfusion, was significantly lower overall in subjects with CAD compared to controls (*p* < .001). (c) PBR did not differ between males with CAD and controls. (d) RBC filling percentage was significantly lower in males with CAD compared to male controls (*p* < .001). (e) PBR was significantly greater in women with CAD compared to their controls (*p* = .04). (f) RBC filling percentage is significantly lower in females with CAD compared to control women (*p* < .001). Data are presented using Tukey outlier box plot and violin plot with in blue the controls and CAD in red

The mean PBR of sublingual microvessels of 5–25 µm diameter was not significantly greater in the CAD group compared to the control group (*p* = .08; Figure 2a, Table 2). Differences remained nonsignificant after adjusting for age, race, BMI, hypertension, and hyperlipidemia (*p* = .32, Table 3). However, a significantly greater PBR (reduced barrier function) was observed in women with CAD compared to control women (*p* = .04) (Figure 2e; Table 2). This difference remained significant following adjustment for potential confounders (*p* = .04, Table 3). When microvessels were stratified by size, there were trends toward greater PBRs in microvessels of 10–19 µm diameter (*p* = .07) and 20–25 µm diameter (*p* = .05) in women with CAD. After adjustment for confounding, there was a significantly greater PBR, and hence reduced glycocalyx barrier properties, in the smallest diameter (5–9 µm) microvessels (*p* = .03) and in 20–25 µm microvessels (*p* = .04) in women with CAD compared to their controls; in microvessels of 10–19 µm diameter, there was a trend toward a greater PBR (*p* = .08) (Table 3). In contrast to females, we found no significant difference in PBR values between male CAD cases and male controls (Figure 2c; Table 2).

**Table 2:** PBR and RBC filling by CAD status

	Control	CAD	*p*‐Value
All patients			
# Patients	63	52	
PBR (5–25 µm)	2.06 ± 0.26	2.15 ± 0.32	.08
PBR (5–9 µm)	1.20 ± 0.11	1.23 ± 0.12	.21
PBR (10–19 µm)	2.22 ± 0.32	2.32 ± 0.38	.15
PBR (20–25 µm)	2.50 ± 0.41	2.65 ± 0.47	.07
RBC filling %	77.71 ± 4.77	73.12 ± 6.43	<.001
Valid density (µm/mm^2^)	7,110 ± 2,299	6,573 ± 2,235	.21
Males			
# Patients	22	39	
PBR (5–25 µm)	2.00 ± 0.25	2.12 ± 0.32	.17
PBR (5–9 µm)	1.18 ± 0.09	1.22 ± 0.13	.29
PBR (10–19 µm)	2.16 ± 0.30	2.27 ± 0.37	.24
PBR (20–25 µm)	2.43 ± 0.44	2.60 ± 0.45	.16
RBC filling %	79.21 ± 3.98	74.00 ± 6.52	.001
Valid density (µm/mm^2^)	7,172 ± 1945	6,734 ± 2,227	.44
Females			
# Patients	41	13	
PBR (5–25 µm)	2.09 ± 0.26	2.27 ± 0.32	.04
PBR (5–9 µm)	1.21 ± 0.12	1.26 ± 0.11	.19
PBR (10–19 µm)	2.25 ± 0.33	2.46 ± 0.39	.07
PBR (20–25 µm)	2.54 ± 0.39	2.81 ± 0.52	.05
RBC filling %	76.90 ± 5.00	70.47 ± 5.53	<.001
Valid density (µm/mm^2^)	7,078 ± 2,490	6,090 ± 2,276	.21

Note

All values shown as mean (*SD*); two‐sample *t* test was used to test for group differences.

**Table 3:** CAD patients versus controls

	Unadjusted			Adjusted^a^		
	Beta	95% CI	*p*‐Value	Beta	95% CI	*p*‐Value
All patients						
PBR (5–25 µm)	0.10	(−0.01, 0.20)	.08	0.07	(−0.06, 0.20)	.32
PBR (5–9 µm)	0.03	(−0.01, 0.07)	.21	0.05	(0.00, 0.11)	.05
PBR (10–19 µm)	0.10	(−0.03, 0.23)	.15	0.07	(−0.09, 0.22)	.43
PBR (20–25 µm)	0.15	(−0.01, 0.31)	.07	0.09	(−0.10, 0.29)	.37
RBC filling %	−0.05	(−0.06, −0.03)	<.01	−0.05	(−0.07, −0.02)	<.01
Valid density (µm/mm^2^)	−53.72	(−137, 29.6)	.21	−23.17	(−126, 80.2)	.66
Males						
PBR (5–25 µm)	0.11	(−0.04, 0.27)	.17	0.02	(−0.16, 0.21)	.84
PBR (5–9 µm)	0.03	(−0.02, 0.09)	.29	0.04	(−0.03, 0.11)	.33
PBR (10–19 µm)	0.11	(−0.07, 0.29)	.24	0.01	(−0.20, 0.23)	.90
PBR (20–25 µm)	0.17	(−0.06, 0.40)	.16	0.01	(−0.26, 0.29)	.92
RBC filling %	−0.05	(−0.08, −0.02)	<.01	−0.05	(−0.08, −0.01)	.01
Valid density (µm/mm^2^)	−43.75	(−155, 67.6)	.44	2.50	(−135,140)	.97
Females						
PBR (5–25 µm)	0.19	(0.01, 0.36)	.04	0.23	(0.02, 0.43)	.04
PBR (5–9 µm)	0.05	(−0.02, 0.12)	.19	0.10	(0.01, 0.18)	.03
PBR (10–19 µm)	0.21	(−0.00, 0.42)	.07	0.23	(−0.02, 0.49)	.08
PBR (20–25 µm)	0.27	(0.003, 0.53)	.05	0.34	(0.03, 0.65)	.04
RBC filling %	−0.06	(−0.09, −0.03)	<.01	−0.07	(−0.10, −0.03)	<.01
Valid density (µm/mm^2^)	−98.76	(−251, 53.6)	.21	−115.70	(−289, 57.6)	.20

a Adjusted for age, race, BMI, hypertension, and hyperlipidemia.

As shown in Figure 2b, RBC filling (percentage of time a microvessel is occupied by RBCs, a measure of microvascular perfusion) was significantly reduced in subjects with CAD compared to controls (*p* < .001) overall. RBC filling percentage was significantly lower in both men (*p* = .001; Figure 2d; Table 2) and women (*p* < .001; Figure 2f; Table 2) with CAD compared to their respective controls. After adjustment for confounding, RBC filling percentage remained significantly reduced in both women and men with CAD compared to their respective controls (Table 3).

Of the 63 controls, 44 were without any known risk factors for cardiovascular disease (37 of 41 female controls and 7 of 22 male controls). The risk factors included cigarette smoking (current, or smoking cessation 0–3 months ago), hypercholesterolemia, hypertension, diabetes mellitus, family history of atherosclerotic disease, and obesity (body mass index >30 kg/m^2^). From medical records, 27 of 39 male CAD cases and 11 of 13 female CAD cases were current or former smokers. We found no effect of smoking history on microvascular glycocalyx variables among the CAD patients, either with or without correction for patient sex, BMI, and presence/absence of hypertension and hyperlipidemia (without correction: *p* = .90 density, *p* = .93 RBC filling percentage, and *p* = .21 PBR; with correction: *p* = .80 density, *p* = .94 RBC filling percentage, and *p* = .26 PBR).

**Figure 3:**
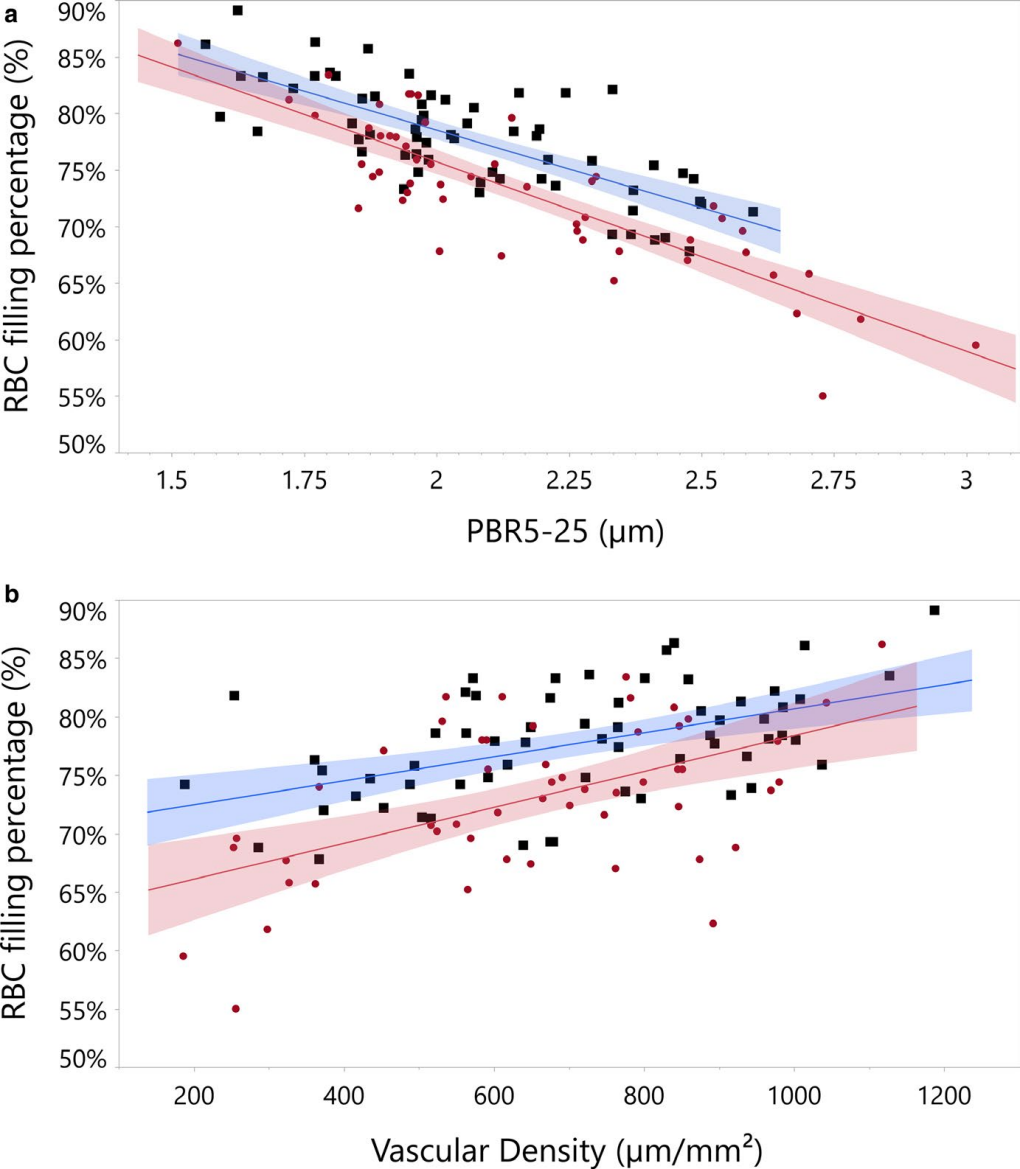
(a) Correlation of perfused boundary region (PBR) and red blood cell (RBC) filling percentage in the sublingual microvasculature of control subjects from the Heart SCORE study (Blue, *r* ^2^ = .56) and subjects with CAD (Red, *r* ^2^ = .71) with fit confidence region. Patients with CAD have a different RBC filling percentage compared to controls at the same PBR values (*p* < .001). The slopes of the curves are not significantly different. (b) Correlation of vascular density and RBC filling percentage in the sublingual microvasculature of control subjects from the Heart SCORE study (Blue, *r* ^2^ = .29) and subjects with CAD (Red, *r* ^2^ = .25) with fit confidence region. Patients with CAD have a higher vascular density compared to controls at similar RBC filling percentages (*p* < .001). However, the slopes of the curves are not significantly different

There was a significant inverse linear relationship between PBR and RBC filling percentage for both males and females (no effect of sex was found regarding this correlation). Patients with CAD had lower RBC filling percentages compared to controls at similar PBR values, consistent with reduced sublingual microvascular perfusion (*p* < .001, Figure 3a). Reductions in glycocalyx barrier function may allow RBCs to enter into the glycocalyx closer to the endothelium. An increase in RBC distribution volume (wider RBC column) results in more space between the RBCs, resulting in a reduced RBC filling percentage. Increases in PBR accompanied by reductions in RBC filling could lead to a reduction in RBC perfused microvessels (reduced functional microvascular density) (Lee et al., 2014); however, in our group, we found no difference in functional microvascular density comparing subjects with CAD to controls (*p* = .21). Differences remained nonsignificant after adjusting for age, race, BMI, hypertension, and hyperlipidemia, and did not differ by sex (Table 3). However, when looking at the controls and CAD participants, we did observe for both groups a significant positive correlation between RBC filling percentage and microvascular density (*r* ^2^ = .25 for patients with CAD and *r* ^2^ = .29 for the control group, Figure 3b). Patients with CAD had a higher vascular density compared to controls at similar RBC filling percentages (*p* < .001; Figure 3b).

## 4 DISCUSSION

Using SDF video microscopy, we found that both males and females with CAD have significantly impaired sublingual microvascular perfusion, measured as the percentage of time microvascular segments are occupied by RBCs. This finding of reduced RBC filling percentage, reflecting a reduced microvascular tube hematocrit, remained significant after adjusting for age, race, BMI, hypertension, and hyperlipidemia. Furthermore, the microvascular RBC hypoperfusion was to some extent independent of glycocalyx barrier function (PBR) as it was reduced in patients with CAD versus controls, even at equivalent levels of PBR.

By SDF imaging, we also evaluated the barrier function of the sublingual microvascular glycocalyx. Here, an increase in PBR indicates deeper penetration of RBCs into the glycocalyx toward the endothelium and therefore reduced glycocalyx barrier function. Females with CAD exhibited a significantly larger overall PBR compared to female controls, whereas males with CAD showed no difference in this measure of glycocalyx barrier function, suggesting a sex‐specific reduction in glycocalyx barrier properties. After excluding any participants with preexisting risk factors for cardiovascular disease, the difference between women with CAD and controls became more pronounced, suggesting that the presence of cardiovascular risk factors might have an effect on glycocalyx barrier function, even in the absence of cardiovascular disease (data not shown).

An intact glycocalyx contributes to the protection of endothelial function throughout the vasculature (Alphonsus & Rodseth, 2014; Reitsma, Slaaf, Vink, & Zandvoort, 2007). Several cardiovascular risk factors, including hyperglycemia, diabetes, and proinflammatory cytokines, are associated with a reduction in microvascular endothelial glycocalyx barrier properties in humans and animal models, consistent with experimental evidence that the glycocalyx defends against vascular insults (van Berg et al., 2003; Constantinescu, Vink, & Spaan, 2001; Constantinescu et al., 2003; Henry & Duling, 2000; Nieuwdorp, Haeften, et al., 2006; Nieuwdorp et al., 2007; Nieuwdorp, Mooij, et al., 2006). Glycocalyx damage has been associated with impaired shear stress‐dependent NO production (Florian et al., 2003; Mochizuki et al., 2003; Pahakis et al., 2007; Tarbell & Pahakis, 2006; Yao, Rabodzey, & Dewey, 2007) and reduction of functional capillary density (Cabrales, Tsai, & Intaglietta, 2007; Marechal et al., 2008; Zuurbier, Demirci, Koeman, Vink, & Ince, 2005). However, the mechanisms underlying endothelial glycocalyx impairments and contribution of these impairments to vascular disease are not fully understood (VanTeeffelen, Brands, Stroes, & Vink, 2007).

Previous data have indicated associations of peripheral microvascular endothelial dysfunction with coronary microvascular dysfunction (Ford et al., 2018). Here, we show that CAD is accompanied by impaired sublingual microvascular perfusion, and that women with CAD, but not men with CAD, have reductions in microvascular glycocalyx barrier function. The magnitude of impaired sublingual microvascular barrier function in our female CAD patients relative to controls was similar to that observed in patients with end‐stage renal disease (Vlahu et al., 2012), lacunar stroke (Martens et al., 2013), ischemic heart disease (Gorshkov et al., 2018), and systemic sclerosis (Machin et al., 2017). A previous study showed that PBR was larger on average in patients with CAD compared to controls (Mulders et al., 2013). However, they made no distinction between males and females, and their patient population was younger (~45 years of age) than those described in our study. A study of patients with age (~65 years) comparable to our study did not find a significant difference between volunteer controls and CAD patients; however, they did not evaluate by sex (Jaarsma et al., 2017). Although our data suggest sex‐specific differences in microvascular glycocalyx barrier function, the mechanisms by which this may occur cannot be inferred from our study. It is noteworthy, however, that coronary microvascular dysfunction is more prevalent in women than in men, suggesting sex related differences underlying these mechanisms (Dean, Cruz, Mehta, & Merz, 2015; Huxley & Kemp, 2018).

Several limitations of our study merit consideration. The control group was not subjected to left heart catheterization to detect or rule out angiographic evidence of coronary artery disease. However, they were recruited from a large cohort study, the Heart SCORE study; after a baseline evaluation, subjects underwent annual visits which included measurements of traditional and emerging CVD risk factors, tabulation of adverse events and assessments of subclinical atherosclerosis. A majority of controls had one or more preexisting risk factors for cardiovascular disease. If anything, however, this would likely bias toward obscuring differences.

The CAD patients had a heterogenous disease presentation, ranging from luminal irregularities to multivessel severe obstructive CAD. Our study was not powered to determine differences in microvascular variables by CAD severity. The racial distribution in the Heart SCORE study, from which our controls were drawn, was roughly representative of Pittsburgh, Pennsylvania (67% White and 23% Black or African American; U.S. Census Bureau, 2010 Census of Population). The number of participants of Black race in our case group recruited at the UPMC Presbyterian University catheterization laboratory, however, was much lower. Studies have shown that people of color are significantly less likely than white people to undergo cardiac catheterization (Bertoni et al., 2005; Schulman et al., 1999). The nature of this bias is not clear.

In summary, the sublingual microvasculature of patients with CAD is characterized by a reduction in perfusion compared to controls. Women, but not men, with CAD exhibited a significantly larger microvascular PBR, indicting a reduction in glycocalyx barrier function suggesting sex dependency. These data suggest that SDF imaging of the sublingual glycocalyx might have potential as a rapid, noninvasive, portable technique to interrogate the pathophysiology of coronary artery disease. Further research is needed to determine the role of microvascular disease, including impaired perfusion and glycocalyx barrier properties, in the pathophysiology of CAD, how this may differ by sex and CAD severity, and whether sublingual microvascular variables have utility in predicting CAD.

## CONFLICT OF INTEREST

None.

## References

[phy214351-bib-0001] Aiyer, A. N. , Kip, K. E. , Marroquin, O. C. , Mulukutla, S. R. , Edmundowicz, D. , & Reis, S. E. (2007). Racial differences in coronary artery calcification are not attributed to differences in lipoprotein particle sizes: The Heart Strategies Concentrating on Risk Evaluation (Heart SCORE) Study. American Heart Journal, 153, 328–334. 17239697 10.1016/j.ahj.2006.11.002

[phy214351-bib-0002] Alphonsus, C. S. , & Rodseth, R. N. (2014). The endothelial glycocalyx: A review of the vascular barrier. Anaesthesia, 69, 777–784. 24773303 10.1111/anae.12661

[phy214351-bib-0003] Bambs, C. , Kip, K. E. , Dinga, A. , Mulukutla, S. R. , Aiyer, A. N. , & Reis, S. E. (2011). Low prevalence of "ideal cardiovascular health" in a community‐based population: The heart strategies concentrating on risk evaluation (Heart SCORE) study. Circulation, 123, 850–857. 21321154 10.1161/CIRCULATIONAHA.110.980151 PMC3061396

[phy214351-bib-0004] Bertoni, A. G. , Goonan, K. L. , Bonds, D. E. , Whitt, M. C. , Goff, D. C. Jr , & Brancati, F. L. (2005). Racial and ethnic disparities in cardiac catheterization for acute myocardial infarction in the United States, 1995–2001. Journal of the National Medical Association, 97, 317–323. 15779495 PMC2568623

[phy214351-bib-0005] Cabrales, P. , Tsai, A. G. , & Intaglietta, M. (2007). Perfluorocarbon in microcirculation during ischemia reperfusion. Journal of the American College of Surgeons, 204, 225–235. 10.1016/j.jamcollsurg.2006.11.007 17254926

[phy214351-bib-0006] Constantinescu, A. A. , Vink, H. , & Spaan, J. A. (2001). Elevated capillary tube hematocrit reflects degradation of endothelial cell glycocalyx by oxidized LDL. American Journal of Physiology‐Heart and Circulatory Physiology, 280, H1051–H1057. 10.1152/ajpheart.2001.280.3.H1051 11179046

[phy214351-bib-0007] Constantinescu, A. A. , Vink, H. , & Spaan, J. A. (2003). Endothelial cell glycocalyx modulates immobilization of leukocytes at the endothelial surface. Arteriosclerosis, Thrombosis, and Vascular Biology, 23, 1541–1547. 10.1161/01.ATV.0000085630.24353.3D 12855481

[phy214351-bib-0008] Dane, M. J. , Khairoun, M. , Lee, D. H. , van den Berg, B. M. , Eskens, B. J. , Boels, M. G. , … Rabelink, T. J. (2014). Association of kidney function with changes in the endothelial surface layer. Clinical Journal of the American Society of Nephrology, 9, 698–704. 10.2215/CJN.08160813 24458084 PMC3974363

[phy214351-bib-0009] Dane, M. J. , van den Berg, B. M. , Lee, D. H. , Boels, M. G. , Tiemeier, G. L. , Avramut, M. C. , … Rabelink, T. J. (2015). A microscopic view on the renal endothelial glycocalyx. American Journal of Physiology‐Renal Physiology, 308, F956–F966. 10.1152/ajprenal.00532.2014 25673809

[phy214351-bib-0010] Dean, J. , Cruz, S. D. , Mehta, P. K. , & Merz, C. N. (2015). Coronary microvascular dysfunction: Sex‐specific risk, diagnosis, and therapy. Nature Reviews Cardiology, 12, 406–414. 26011377 10.1038/nrcardio.2015.72

[phy214351-bib-0011] Florian, J. A. , Kosky, J. R. , Ainslie, K. , Pang, Z. , Dull, R. O. , & Tarbell, J. M. (2003). Heparan sulfate proteoglycan is a mechanosensor on endothelial cells. Circulation Research, 93, e136–e142. 10.1161/01.RES.0000101744.47866.D5 14563712

[phy214351-bib-0012] Ford, T. J. , Rocchiccioli, P. , Good, R. , McEntegart, M. , Eteiba, H. , Watkins, S. , … Berry, C. (2018). Systemic microvascular dysfunction in microvascular and vasospastic angina. European Heart Journal, 39, 4086–4097. 10.1093/eurheartj/ehy529 30165438 PMC6284165

[phy214351-bib-0013] Gori, T. (2018). Endothelial function: A short guide for the interventional cardiologist. International Journal of Molecular Sciences, 19(12), 3838 10.3390/ijms19123838 PMC6320818 30513819

[phy214351-bib-0014] Gorshkov, A. Y. , Klimushina, M. V. , Boytsov, S. A. , Kots, A. Y. , & Gumanova, N. G. (2018). Increase in perfused boundary region of endothelial glycocalyx is associated with higher prevalence of ischemic heart disease and lesions of microcirculation and vascular wall. Microcirculation, 25, e12454 10.1111/micc.12454 29608790

[phy214351-bib-0015] Henry, C. B. , & Duling, B. R. (1999). Permeation of the luminal capillary glycocalyx is determined by hyaluronan. American Journal of Physiology‐Heart and Circulatory Physiology, 277, H508–H514. 10.1152/ajpheart.1999.277.2.H508 10444475

[phy214351-bib-0016] Henry, C. B. , & Duling, B. R. (2000). TNF‐alpha increases entry of macromolecules into luminal endothelial cell glycocalyx. American Journal of Physiology Heart and Circulatory Physiology, 279, H2815–H2823. 11087236 10.1152/ajpheart.2000.279.6.H2815

[phy214351-bib-0017] Hermansson, J. , & Kahan, T. (2018). Systematic review of validity assessments of framingham risk score results in health economic modelling of lipid‐modifying therapies in Europe. Pharmacoeconomics, 36, 205–213. 10.1007/s40273-017-0578-1 29079929 PMC5805819

[phy214351-bib-0018] Huxley, V. H. , & Kemp, S. S. (2018). Sex‐specific characteristics of the microcirculation. Advances in Experimental Medicine and Biology, 1065, 307–328. 30051393 10.1007/978-3-319-77932-4_20 PMC6209095

[phy214351-bib-0019] Huxley, V. H. , & Williams, D. A. (2000). Role of a glycocalyx on coronary arteriole permeability to proteins: Evidence from enzyme treatments. American Journal of Physiology Heart and Circulatory Physiology, 278, H1177–H1185. 10749712 10.1152/ajpheart.2000.278.4.H1177

[phy214351-bib-0020] Ikonomidis, I. , Pavlidis, G. , Lambadiari, V. , Kousathana, F. , Varoudi, M. , Spanoudi, F. , … Lekakis, J. (2017). Early detection of left ventricular dysfunction in first‐degree relatives of diabetic patients by myocardial deformation imaging: The role of endothelial glycocalyx damage. International Journal of Cardiology, 233, 105–112. 28096045 10.1016/j.ijcard.2017.01.056

[phy214351-bib-0021] Jaarsma, C. , Vink, H. , van Haare, J. , Bekkers, S. , van Rooijen, B. D. , Backes, W. H. , … Schalla, S. (2017). Non‐invasive assessment of microvascular dysfunction in patients with microvascular angina. International Journal of Cardiology, 248, 433–439. 10.1016/j.ijcard.2017.05.010 28733074

[phy214351-bib-0022] Lee, D. H. , Dane, M. J. , van den Berg, B. M. , Boels, M. G. S. , van Teeffelen, J. W., … Rabelink, T. J. (2014). Deeper penetration of erythrocytes into the endothelial glycocalyx is associated with impaired microvascular perfusion. PLoS ONE, 9, e96477 10.1371/journal.pone.0096477 24816787 PMC4015985

[phy214351-bib-0023] Machin, D. R. , Gates, P. E. , Vink, H. , Frech, T. M. , & Donato, A. J. (2017). Automated measurement of microvascular function reveals dysfunction in systemic sclerosis: A cross‐sectional study. Journal of Rheumatology, 44, 1603–1611. 28916547 10.3899/jrheum.170120 PMC5668162

[phy214351-bib-0024] Marechal, X. , Favory, R. , Joulin, O. , Montaigne, D. , Hassoun, S. , Decoster, B. , … Neviere, R. (2008). Endothelial glycocalyx damage during endotoxemia coincides with microcirculatory dysfunction and vascular oxidative stress. Shock, 29, 572–576. 10.1097/shk.0b013e318157e926 18414231

[phy214351-bib-0025] Martens, R. J. , Vink, H. , van Oostenbrugge, R. J. , & Staals, J. (2013). Sublingual microvascular glycocalyx dimensions in lacunar stroke patients. Cerebrovascular Diseases, 35, 451–454. 10.1159/000348854 23735841

[phy214351-bib-0026] Matsuzawa, Y. , & Lerman, A. (2014). Endothelial dysfunction and coronary artery disease: Assessment, prognosis, and treatment. Coronary Artery Disease, 25, 713–724. 25365643 10.1097/MCA.0000000000000178 PMC4220301

[phy214351-bib-0027] Miranda, C. H. , de Carvalho, B. M. , Schmidt, A. , Marin‐Neto, J. A. , & Pazin‐Filho, A. (2016). Evaluation of the endothelial glycocalyx damage in patients with acute coronary syndrome. Atherosclerosis, 247, 184–188. 10.1016/j.atherosclerosis.2016.02.023 26926597

[phy214351-bib-0028] Mochizuki, S. , Vink, H. , Hiramatsu, O. , Kajita, T. , Shigeto, F. , Spaan, J. A. , & Kajiya, F. (2003). Role of hyaluronic acid glycosaminoglycans in shear‐induced endothelium‐derived nitric oxide release. American Journal of Physiology‐Heart and Circulatory Physiology, 285, H722–H726. 10.1152/ajpheart.00691.2002 12730059

[phy214351-bib-0029] Mulders, T. A. , Nieuwdorp, M. , Stroes, E. S. , Vink, H. , & Pinto‐Sietsma, S. J. (2013). Non‐invasive assessment of microvascular dysfunction in families with premature coronary artery disease. International Journal of Cardiology, 168, 5026–5028. 10.1016/j.ijcard.2013.07.166 23968713

[phy214351-bib-0030] Mulukutla, S. R. , Venkitachalam, L. , Bambs, C. , Kip, K. E. , Aiyer, A. , Marroquin, O. C. , & Reis, S. E. (2010). Black race is associated with digital artery endothelial dysfunction: Results from the Heart SCORE study. European Heart Journal, 31, 2808–2815. 20736241 10.1093/eurheartj/ehq295

[phy214351-bib-0031] Nieuwdorp, M. , Holleman, F. , de Groot, E. , Vink, H. , Gort, J. , Kontush, A. , … Stroes, E. S. (2007). Perturbation of hyaluronan metabolism predisposes patients with type 1 diabetes mellitus to atherosclerosis. Diabetologia, 50, 1288–1293. 10.1007/s00125-007-0666-4 17415544 PMC1914278

[phy214351-bib-0032] Nieuwdorp, M. , Mooij, H. L. , Kroon, J. , Atasever, B. , Spaan, J. A. , Ince, C. , … Vink, H. (2006). Endothelial glycocalyx damage coincides with microalbuminuria in type 1 diabetes. Diabetes, 55, 1127–1132. 10.2337/diabetes.55.04.06.db05-1619 16567538

[phy214351-bib-0033] Nieuwdorp, M. , van Haeften, T. W. , Gouverneur, M. C. , Mooij, H. L. , van Lieshout, M. H. , Levi, M. , … Stroes, E. S. (2006). Loss of endothelial glycocalyx during acute hyperglycemia coincides with endothelial dysfunction and coagulation activation in vivo. Diabetes, 55, 480–486. 10.2337/diabetes.55.02.06.db05-1103 16443784

[phy214351-bib-0034] Olafiranye, O. , Kip, K. E. , Rhinehart, Z. , Mulukutla, S. R. , Aiyer, A. , Strollo, P. J. , & Reis, S. E. (2015). Impact of race and obesity on arterial endothelial dysfunction associated with sleep apnea: Results from the Heart SCORE study. International Journal of Cardiology, 201, 476–478. 26313870 10.1016/j.ijcard.2015.08.098

[phy214351-bib-0035] Pahakis, M. Y. , Kosky, J. R. , Dull, R. O. , & Tarbell, J. M. (2007). The role of endothelial glycocalyx components in mechanotransduction of fluid shear stress. Biochemical and Biophysical Research Communications, 355, 228–233. 10.1016/j.bbrc.2007.01.137 17291452 PMC1847369

[phy214351-bib-0036] Reitsma, S. , Slaaf, D. W. , Vink, H. , & van Zandvoort, M. A. (2007). and oude Egbrink MG. The endothelial glycocalyx: Composition, functions, and visualization. Pflugers Archiv: European Journal of Physiology, 454, 345–359. 17256154 10.1007/s00424-007-0212-8 PMC1915585

[phy214351-bib-0037] Schulman, K. A. , Berlin, J. A. , Harless, W. , Kerner, J. F. , Sistrunk, S. , Gersh, B. J. , … Escarce, J. J. (1999). The effect of race and sex on physicians' recommendations for cardiac catheterization. The New England Journal of Medicine, 340, 618–626. 10.1056/NEJM199902253400806 10029647

[phy214351-bib-0038] Tarbell, J. M. , & Pahakis, M. Y. (2006). Mechanotransduction and the glycocalyx. Journal of Internal Medicine, 259, 339–350. 10.1111/j.1365-2796.2006.01620.x 16594902

[phy214351-bib-0039] van den Berg, B. M. , Spaan, J. A. , Rolf, T. M. , & Vink, H. (2006). Atherogenic region and diet diminish glycocalyx dimension and increase intima‐to‐media ratios at murine carotid artery bifurcation. American Journal of Physiology‐Heart and Circulatory Physiology, 290, H915–H920. 10.1152/ajpheart.00051.2005 16155109

[phy214351-bib-0040] van den Berg, B. M. , Vink, H. , & Spaan, J. A. (2003). The endothelial glycocalyx protects against myocardial edema. Circulation Research, 92, 592–594. 10.1161/01.RES.0000065917.53950.75 12637366

[phy214351-bib-0041] VanTeeffelen, J. W. , Brands, J. , Stroes, E. S. , & Vink, H. (2007). Endothelial glycocalyx: Sweet shield of blood vessels. Trends in Cardiovascular Medicine, 17, 101–105. 17418372 10.1016/j.tcm.2007.02.002

[phy214351-bib-0042] Vlahu, C. A. , Lemkes, B. A. , Struijk, D. G. , Koopman, M. G. , Krediet, R. T. , & Vink, H. (2012). Damage of the endothelial glycocalyx in dialysis patients. Journal of the American Society of Nephrology, 23, 1900–1908. 10.1681/ASN.2011121181 23085635 PMC3482728

[phy214351-bib-0043] Wolfson, J. , Vock, D. M. , Bandyopadhyay, S. , Kottke, T. , Vazquez‐Benitez, G. , Johnson, P. , … O'Connor, P. J. (2017). Use and customization of risk scores for predicting cardiovascular events using electronic health record data. Journal of the American Heart Association, 6(4), 1–11. 10.1161/JAHA.116.003670. PMC5532984 28438733

[phy214351-bib-0044] Xue, X.‐J. , Jiang, Y. , Chen, L. , & Chen, S.‐L. (2018). Relationship between the endothelial glycocalyx and the extent of coronary atherosclerosis. Microcirculation, 25(8), e12504 10.1111/micc.12504 30192430

[phy214351-bib-0045] Yao, Y. , Rabodzey, A. , & Dewey, C. F. Jr . (2007). Glycocalyx modulates the motility and proliferative response of vascular endothelium to fluid shear stress. American Journal of Physiology‐Heart and Circulatory Physiology, 293, H1023–H1030. 10.1152/ajpheart.00162.2007 17468337

[phy214351-bib-0046] Zuurbier, C. J. , Demirci, C. , Koeman, A. , Vink, H. , & Ince, C. (2005). Short‐term hyperglycemia increases endothelial glycocalyx permeability and acutely decreases lineal density of capillaries with flowing red blood cells. Journal of Applied Physiology, 99, 1471–1476. 10.1152/japplphysiol.00436.2005 16024521

